# Variance adjusted weighted UniFrac: a powerful beta diversity measure for comparing communities based on phylogeny

**DOI:** 10.1186/1471-2105-12-118

**Published:** 2011-04-25

**Authors:** Qin Chang, Yihui Luan, Fengzhu Sun

**Affiliations:** 1 School of Mathematics, Shandong University, Jinan, Shandong 250100, PR China; 2 TNLIST/Department of Automation, Tsinghua University, Beijing 100084, PR China; 3 Molecular and Computational Biology Program, University of Southern California, Los Angeles, CA 90089-2910, USA

## Abstract

**Background:**

Beta diversity, which involves the assessment of differences between communities, is an important problem in ecological studies. Many statistical methods have been developed to quantify beta diversity, and among them, UniFrac and weighted-UniFrac (W-UniFrac) are widely used. The W-UniFrac is a weighted sum of branch lengths in a phylogenetic tree of the sequences from the communities. However, W-UniFrac does not consider the variation of the weights under random sampling resulting in less power detecting the differences between communities.

**Results:**

We develop a new statistic termed variance adjusted weighted UniFrac (VAW-UniFrac) to compare two communities based on the phylogenetic relationships of the individuals. The VAW-UniFrac is used to test if the two communities are different. To test the power of VAW-UniFrac, we first ran a series of simulations which revealed that it always outperforms W-UniFrac, as well as UniFrac when the individuals are not uniformly distributed. Next, all three methods were applied to analyze three large 16S rRNA sequence collections, including human skin bacteria, mouse gut microbial communities, microbial communities from hypersaline soil and sediments, and a tropical forest census data. Both simulations and applications to real data show that VAW-UniFrac can satisfactorily measure differences between communities, considering not only the species composition but also abundance information.

**Conclusions:**

VAW-UniFrac can recover biological insights that cannot be revealed by other beta diversity measures, and it provides a novel alternative for comparing communities.

## Background

The assessment of differences between communities is an important problem in ecological studies. By comparing the compositions of natural communities from different environments, locations or time periods, we can learn how specific factors affect community assembly and how species or individuals associate with each other [1-3]. The development of next-generation high-throughput sequencers, such as the 454 Life Sciences Genome Sequencer FLX System, the Illumina 1G Genome Analysis System, and Applied Biosystems SOLiD Sequencing, has profoundly changed our approaches to ecological studies. With the rapid development of sequencing technologies, it is now possible to sequence a particular gene, such as 16S rRNA sequences, at very high depth without culturing [2,4-6]. The new sequencing technologies also make it possible to efficiently and economically sequence the whole metagenome within a community [7,8]. These techniques have revealed high microbial diversity present in the ocean, soil and human tissues.

Many statistics have been proposed to compare communities based on genomic sequence data of a specific gene sampled from the communities. These include LIBSHUFF [9], ∫-LIBSHUFF [10], analysis of molecular variance (AMOVA) [11,12], and homogeneity of molecular variance (HOMOVA) [13]. They mainly depend on the distances or similarities between sequences within the same community and between different communities. Other statistical methods for community comparison depend on a specific phylogenetic tree of the sequences and the tree can be either pre-defined or inferred from the genomic sequences. Such statistics include the parsimony test [14,15], UniFrac [16], and weighted UniFrac (W-UniFrac) [17]. In the parsimony test, each sequence is labeled according to the community it belongs to and then the parsimony score, the number of minimal changes along the tree necessary to explain all the labels of the sequences, is calculated according to Fitch's parsimony algorithm [18]. The statistical significance of the parsimony score has been evaluated using two different randomization procedures. The first randomization procedure is to randomize the tree for the sequences [14] and the second procedure is to randomize the labels of the sequences conditional on the tree [16]. These two randomization procedures test for different hypotheses. The first randomization procedure evaluates whether the sequences from the communities cluster randomly and the second procedure evaluates if the sequences are randomly distributed on the leaves of the given phylogenetic tree.

Lozupone et al. proposed a novel statistical method termed UniFrac [16] and a weighted UniFrac (W-UniFrac) [17] to test if two communities are significantly different based on a phylogenetic tree. They have been widely applied to numerous recent studies to compare microbial communities, and significant biological insights have been obtained [2,4,19]. The procedures for calculating UniFrac and W-UniFrac can be briefly described as follows. A phylogenetic tree composed of sequences from all the communities is first constructed using a phylogenetic analysis tool such as PHYLIP [20]. Similar as in the parsimony test, each sequence is labeled according to the community it comes from. Then UniFrac measures the distance between communities by the fraction of length of the tree branches that lead to descendants from each single community, but not from both communities [16]. The W-UniFrac takes abundance information into consideration and weights each branch length by the difference of the fractions of sequences belonging to the branch for the two communities [17]. The significance of both tests are evaluated by randomizing the labels of the sequences. Using this randomization procedure, both UniFrac and W-UniFrac test the hypothesis that the sequence labels are random along the leaves of the tree.

Despite the many studies on statistical methods to compare communities, there had been some confusions about the hypotheses being tested for the different statistics. Schloss [21] addressed this important issue using simulations. It was shown that AMOVA can be used to test if sequences from the different communities have the same mean (center) and HOMOVA can be used to evaluate if the variations within the communities are the same. On the other hand, the parsimony test, UniFrac and W-UniFrac are valid for evaluating the general hypothesis that the communities are the same.

Note that in W-UniFrac the length of a branch is weighted by the difference of relative abundances of the two communities for that branch of the tree. Under the null hypothesis that the two communities are the same, the weights for the different branches in W-UniFrac have difference variances and we provide a formula for calculating the variance in this paper. Based on the variance formula, we propose a new weighting scheme for the branch length in W-UniFrac by taking the variation of the weight into consideration. The new resulting statistic is termed Variance Adjusted Weighted UniFrac (VAW-UniFrac). The statistical significance of VAW-UniFrac is evaluated by randomizing the labels of the sequences along the leaves of the tree. Similar to UniFrac and W-UniFrac, the VAW-UniFrac can be used to evaluate if two communities are different. More precisely, it tests the hypothesis that the sequences from the communities are randomly distributed along the leaves of the tree. To study the power of this new statistic, we first carried out simulation studies similar to that in [21] to detect differences between communities based on UniFrac, W-UniFrac and VAW-UniFrac. The power of VAW-UniFrac is always higher than that of W-UniFrac. When the individuals are uniformly distributed in both communities, UniFrac can be more powerful than both W-UniFrac and VAW-UniFrac. However, when the individuals are not uniformly distributed, VAW-UniFrac is more powerful than both UniFrac and W-UniFrac. We also utilized UniFrac, W-UniFrac, and VAW-UniFrac in a reanalysis of four different real datasets, including three 16S rRNA sequence collections from different studies and one forest census data. Since VAW-UniFrac demonstrated a capacity to gain novel biological insights beyond that of either UniFrac or W-UniFrac, we concluded that VAW-UniFrac offers a highly useful alternative approach for comparing communities.

## Methods

### UniFrac and W-UniFrac

Given a phylogenetic tree composed of all individuals from two communities, UniFrac distance [16] is defined as the fraction of the branch length that leads to descendants from each single community, but not from both communities. Thus, it captures the total amount of evolutionary history that is unique to each community, presumably reflecting adaptation to one environment, but non-adaptation to the other. W-UniFrac [17] weights each branch length by the abundance differences of the branch along the tree of the communities. UniFrac focuses on the presence/absence of species in communities, not the abundance levels of these species. Thus, the UniFrac distance between two communities with the same species, but different species abundances, is zero, while the W-UniFrac distance is not zero. Therefore, if the abundance differences are of interest, W-UniFrac should be used. The W-UniFrac distance can be calculated using the following equation,

In the numerator, *n *is the number of branches in the tree, *b_i _*is the length of branch *i*, *A_i _*and *B_i _*are the numbers of individuals that descend from branch *i *in communities A and B, respectively, and *A_T _*and *B_T _*are the total numbers of individuals in communities A and B, respectively. In the denominator, *n' *is the number of different individuals in the two communities, *d_j _*is the distance from the root to individual *j*, while *α_j _*and *β_j _*are the numbers of times the sequences were observed in communities A and B, respectively (all the above numbers of individuals should be counted with multiplicity, except *n'*). The same annotation will be used in the rest of the paper.

Note that

Therefore, the denominator of *WU *is always larger than, or equal to, the numerator. Equality holds when either *A_i _*= 0 or *B_i _*= 0, which means that the two communities are totally separated. The numerators of both UniFrac and W-UniFrac can be written as

In W-UniFrac,

and in UniFrac,

Where , if there are sequences that descend from branch *i *in communities A, and  otherwise. The case is similar for . It means that the UniFrac metric can be represented by

### A novel variance adjusted weighted UniFrac (VAW-UniFrac) for comparing communities

From the definition of W-UniFrac given above, we note that it does not consider the variance of the weight  for the *i*-th branch length assuming that the sequence labels are randomly distributed along the leaves of the tree. By ignoring the variance of *ω_i _*in W-UniFrac, the true relationships between communities may not be well characterized. Hence, we propose to adjust the weight *ω_i _*as follows. Given individuals from two communities, A and B, we first generate a phylogenetic tree composed of all the *A_T _*+ *B_T _*individuals in communities as leaves. Each leaf is labeled "A" or "B" to represent the community from which it comes. We test the hypothesis that the labels of the individuals are randomly distributed on the phylogenetic tree.

Consider the *i*-th branch of the phylogenetic tree. Let *m_i _*= *A_i _*+ *B_i _*be the total number of individuals belonging to the *i*-th branch. Let *m *= *A_T _*+ *B_T _*. We randomly choose *A_T _*individuals from the total of *m *individuals, label them as being from community A and label the other leaves as being from community B. Then, *A_i_*, the number of individuals in community A that belong to the *i*-th branch, is hypergeometric with parameters (*m_i_*, *m*, *A_T _*) under the null hypothesis. That is

Therefore,

Let

Under the null hypothesis that the *A_T _*individuals are randomly sampled from the *m *individuals, we have

and

From the above derivation, we propose the following variance adjusted weight (VAW) for the length of the *i*-th branch of the tree,

We standardize the resulting statistic so that its value is between 0 and 1. The final VAW-UniFrac is defined as

In addition to the statistic *T *, we also consider a variation by taking the weight as  which gives

Similar to UniFrac and W-UniFrac, the VAW-UniFrac aims to test if the two communities are different and, more specifically, if the sequences are randomly distributed along the leaves of the tree. The statistical significance of VAW-UniFrac is evaluated by randomizing the labels of the sequences. We are interested in which methods, including UniFrac, W-UniFrac (*WU*), VAW-UniFrac (*T *) or *SqT *, are more powerful in detecting the relationship between two communities if they are related.

### Simulation studies to compare the power of the statistics for detecting the relationships between communities

Schloss [21] evaluated the power of several different statistics for comparing the relationships between communities and studied the validity of the different statistics for testing various hypotheses. These statistical techniques included TreeClimber [15], UniFrac [16], W-UniFrac [17], *∫*-LIBSHUFF [10], AMOVA [22], and HOMOVA [23]. In our study, similar simulation approaches are used to compare the power of UniFrac, W-UniFrac, as well as VAW-UniFrac and its variation SqT. Our objective is to understand which statistics are the most powerful and under what conditions. Since the simulation approaches are similar to those in [21], we only present a very brief description.

In the simulations, a community was represented by the interior of a circle or an ellipse with a certain density. Changing the overlap between circles (ellipses) or the distribution patterns of samples in circles (ellipses) represented changing the differences between the communities. The maximum distance between any two points in one community was designed to be 0.3 units according to the distance between sequences from different phyla [21]. Three classes of overlapping patterns were simulated. In the first class, the two communities were represented as circles, and points were uniformly sampled from each circle. The different overlapping patterns were obtained by changing the center and the radius of one circle. In the second class, the two communities were represented as ellipses, and points were uniformly sampled from each ellipse. The overlapping patterns were obtained by rotating one of the ellipses. In the third class, the two communities were represented by the same circle. One community was uniformly sampled from the circle, and the distribution of the points in the other community was not uniform.

For one comparison, we first sampled 200 points from each community, resulting in a total of 400 points. Second, Euclidean distances among all 400 points were calculated. Third, a phylogeny tree was generated based on this distance matrix using the neighbor joining method in the neighbor program in PHYLIP [20]. Fourth, the four statistics, UniFrac, W-UniFrac, VAW-UniFrac(T), and SqT, could then be calculated. Fifth, the labels of the 400 points were randomized 1000 times with the tree topology unchanged, and the corresponding four statistics were calculated for each randomized dataset. Finally, a P-value was calculated by the proportion of randomizations which result in statistics that are either equal to, or greater than, the original statistic. P-values less than 0.05 were considered significant. Therefore, after 1000 independent samplings, a proportion of significant P-values was obtained, representing the type 1 error rate when two communities were the same and the statistical power when the communities were different.

### Applications to four real data sets

We applied UniFrac, W-UniFrac and VAW-UniFrac to reanalyze three datasets consisting of 16S rRNA sequences and one tropical forest census data. First, Costello et al. [4] investigated how environmental factors and foreign transplants shape skin bacterial communities. Plots on two skin sites of volunteers, both forehead and left volar forearm, were first disinfected, then inoculated with foreign microbiotas from other tissues, and, finally, followed over 2, 4 and 8 hours. The data were downloaded from the European Read Archive [ERA:ERA000159]. Second, Ley et al. [2] studied the effects of obesity and kinship on mouse distal intestinal microbial communities based on 16S rRNA gene sequence collections. They sampled 16S rRNA gene sequences obtained from the distal ceca of 19 mice, including 3 heterozygous (ob/+) mothers (M1, M2, and M3) and their 16 offspring with all three possible genotypes (obese ob/ob mice, lean ob/+ and wild-type +/+ mice). The final data, including all the sequences, ARB alignment [24] and phylogenetic tree are publicly available at http://gordonlab.wustl.edu/mice. Third, Hollister et al. [25] studied the microbial diversity of soil and sediments using both Sanger sequencing and pyrosequencing. Samples were collected at eight locations (T3-0, T3-65, T3-130, T3-195 T3-260, T3-325, T3-390, and T3-455) along a geographical transect from the shoreline of a hypersaline lake and lakebed. Point T3-0 is the terrestrial end of the transect, while point T3-455 is the aquatic end. For each sample, both Sanger sequencing and pyrosequencing were performed. A total of 39590 16S rRNA sequences were generated through 454 sequencing, and 1693 16S rRNA sequences were generated through cloning and single-pass Sanger sequencing. The pyrosequencing libraries ranged in size from 1403 sequences at site T3-0 to 6745 sequences at site T3-325. The Sanger clone collections ranged in size from 185 sequences at T3-0 and T3-130 to 230 sequences at T3-390 [25]. All the sequences were downloaded from NCBI [GenBank:CQ893028-CQ894720, SRA:SRA009427.2]. The fourth dataset involves tropical forest census data in three plots across a precipitation gradient in central Panama [26-28]. The Cocoli 4-ha plot is located in a dry, semi-deciduous forest on the Pacific side, and it has 3 census data: 1994, 1997, and 1998. The 50-ha BCI plot is located in the tropical moist forest of Barro Colorado Island (BCI) in central Panama, and it has 6 census data: 1981-1983, 1985, 1990, 1995, 2000, and 2005. The third plot is the Sherman 5.6-ha plot, the wettest of the three, located near the Atlantic coast, 55 km northwest of the Cocoli site. This plot has three census data: 1996, late 1997 to early 1998, and 1999. These census data recorded all free-standing woody plants with stem diameter 1 cm or above in the plots [26]. Different from the above applications, the original abundance information for each species was available.

For each dataset, we first calculated the distances between each pair of communities using the three statistics: UniFrac, W-UniFrac and VAW-UniFrac. Then the unweighted pair group method with arithmetic averages (UPGMA) clustering [29] was used to cluster the communities. The resulting clusters were then analyzed based on the characteristics of the individuals in each cluster. Principal coordinate analysis (PCoA) [30] was also used to project the communities into a two-dimensional plane determined by the first two principal coordinates to determine whether communities with similar characteristics tend to cluster together.

## Results and Discussion

In order to study our new methods and compare their performance to UniFrac and W-UniFrac, we carried out simulation studies according to the simulation methods developed in [21]. We then used UniFrac, W-UniFrac, and VAW-UniFrac to reanalyze four real datasets, three 16S rRNA sequence collections from different research laboratories, and a tropical forest census dataset.

### Results from Simulation Studies

We carried out three classes of simulations for two communities: 1) both were uniform samples from two circles with different centers and radii; 2) both were uniform samples from two ellipses with different orientations; and 3) one community was a uniform sample, while the other was an uneven sample from the same circle.

#### Simulation 1: communities were uniformly distributed on two circles

In this simulation, communities were represented by circles, and individuals were uniformly sampled from each circle. It is reasonable to assume that the magnitude of a circle's radius can reflect the diversity of the community that it represents. In order to investigate the type 1 error rates and statistical power of the four statistics, UniFrac, W-UniFrac, VAW-UniFrac, and SqT, different levels of overlaps between communities were simulated by changing the distance between two centers, known as offset, and by changing the radius of one of the two circles (Table 1). The radius of one circle corresponding to community A was set at 0.15, and the radius of the other circle corresponding to community B was variably set at 0.15, 0.134 and 0.116. We let the offset be 0, 0.012, 0.024, 0.035, and 0.047, respectively. Table 1 gives the power of the four statistics in detecting the differences between the two communities for type 1 error 0.05. As expected, the type 1 error rates realized for all the four statistics are close to 0.05 (95% confidence interval between 0.036 and 0.064) when the communities are identical, indicating the validity of the methods. The power of all four statistics increases with the offset and the difference between the radii of the two circles (Table 1). The power of UniFrac is superior to the other three in this simulation. However, among the three weighted methods, VAW-UniFrac outperforms W-UniFrac, while VAW-UniFrac performs similarly as SqT.

**Table 1:** Simulated power of four statistics, UniFrac (UniF), W-UniFrac (WUniF), VAW-UniFrac (T), and SqT in Simulation 1.

Radius of B (Overlap)			offset (Overlap)		
	0 (100%)	0.012 (95%)	0.024 (90%)	0.035 (85%)	0.047 (80%)
0.15 (100%)	UniF: 0.050	UniF: 0.224	UniF: 0.898	UniF: 0.999	UniF: 1.000
	WUniF: 0.049	WUniF: 0.152	WUniF: 0.600	WUniF: 0.945	WUniF: 0.999
	T: 0.057	T: 0.208	T: 0.761	T: 0.994	T: 1.000
	SqT: 0.061	SqT: 0.200	SqT: 0.755	SqT: 0.991	SqT: 1.000

0.134 (80%)	UniF: 0.820	UniF: 0.918	UniF: 0.997	UniF: 1.000	UniF: 1.000
	WUniF: 0.124	WUniF: 0.339	WUniF: 0.726	WUniF: 0.973	WUniF: 1.000
	T: 0.311	T: 0.578	T: 0.926	T: 0.997	T: 1.000
	SqT: 0.252	SqT: 0.546	SqT: 0.933	SqT: 1.000	SqT: 1.000

0.116 (60%)	UniF: 1.000	UniF: 1.000	UniF: 1.000	UniF: 1.000	UniF: 1.000
	WUniF: 0.778	WUniF: 0.886	WUniF: 0.974	WUniF: 0.998	WUniF: 1.000
	T: 1.000	T: 1.000	T: 1.000	T: 1.000	T: 1.000
	SqT: 0.999	SqT: 1.000	SqT: 1.000	SqT: 1.000	SqT: 1.000

The first community is represented as a circle with radius 0.15, and the second community is represented as circle "B" with radii 0.15, 0.134, and 0.116, respectively. The offset is the distance between the centers of the two circles. The number of simulations is 1000, and the type 1 error rate is set at 0.05.

#### Simulation 2: communities were uniformly distributed on two ellipses

The second simulation was similar to the first, except that the two simulated communities were uniformly distributed on ellipses, with a length of 0.3 units and a width of 0.15 units. As in Simulation 1, we first made the two ellipses identical and calculated statistics and the resulting P-values. Then different relationships between communities were simulated by pivoting one ellipse while fixing the other (Table 2). The results turned out to be similar to those of Simulation 1. The type 1 error rate realized for each statistic is not significantly different from 0.05. The power of each statistic increases when the differences between communities increase. The relative performance of each statistic is also similar to that of Simulation 1 (Table 2).

**Table 2:** Simulated power of four statistics, UniFrac (UniF), W-UniFrac (WUniF), VAW-UniFrac (T), and SqT in Simulation 2.

Pivot	0°	6°	12°	26°	71°
Power	UniF: 0.050	UniF: 0.185	UniF: 0.822	UniF: 1.000	UniF: 1.000
	WUniF: 0.047	WUniF: 0.126	WUniF: 0.339	WUniF: 0.981	WUniF: 1.000
	T: 0.050	T: 0.166	T: 0.577	T: 1.000	T: 1.000
	SqT: 0.049	SqT: 0.157	SqT: 0.554	SqT: 1.000	SqT: 1.000

The "pivot" is the rotation angle between the long axes of the two ellipses. The number of simulations is 1000, and the type 1 error rate is set at 0.05.

#### Simulation 3: one community was uniformly and the other was unevenly distributed on a circle

In this simulation, we investigated the performance of the four statistics when one community was uniformly distributed and the other community was unevenly distributed on a same circle (Table 3). This simulation mimics situations where two communities share the same membership, but have different distribution patterns. For one community, we drew points uniformly from a circle, while, for the other community, we drew points clumped to the periphery or center of the same circle. When we performed these uneven samplings, the distance from each point to the center was determined by the *c*-th root of a uniform sampling from 0 to 0.15^c ^so that the points we drew would clump to the periphery when *c >*2 and to the center when *c <*2. When *c *= 2, the points were uniformly chosen from the same circle (Table 1, top left corner). In this simulation, statistical power still increases with divergences of communities for all the statistics. When *c >*2, UniFrac outperforms W-UniFrac. On the other hand, when *c <*2, W-UniFrac outperforms UniFrac. VAW-UniFrac outperforms both UniFrac and W-UniFrac in detecting the differences between the two communities by a significant margin for any values of *c*. VAW-UniFrac also performs similarly as SqT.

**Table 3:** Simulated power of four statistics, UniFrac (UniF), W-UniFrac (WUniF), VAW-UniFrac (T), and SqT in Simulation 3.

*c*	6	4	3		1	
Power	UniF: 0.908	UniF: 0.433	UniF: 0.154	UniF: 0.117	UniF: 0.274	UniF: 0.660
	WUniF: 0.802	WUniF: 0.357	WUniF: 0.124	WUniF: 0.150	WUniF: 0.649	WUniF: 1.000
	T: 0.965	T: 0.506	T: 0.160	T: 0.196	T: 0.681	T: 1.000
	SqT: 0.951	SqT: 0.522	SqT: 0.167	SqT: 0.199	SqT: 0.685	SqT: 1.000

The number of simulations is 1000, and the type 1 error rate is set at 0.05.

#### Summary of results from simulation studies

Results of the three simulations reveal that VAW-UniFrac always performs better than W-UniFrac. In Simulation 1 and Simulation 2, UniFrac has the highest statistical power to detect differences between communities. These observations can be explained as follows. In the first two simulations, both communities are uniformly distributed, and there is no need to weight the branch lengths. The inclusion of weights for the branch lengths in both W-UniFrac and VAW-UniFrac introduces more noise into the statistics, resulting in lowered power to detect differences between the communities. In Simulation 3, one of the communities is not uniformly distributed, and since the inclusion of weights can adjust for the uneven distribution, the weighted version is more powerful in general. Because the variance adjusted version takes both the abundance difference and its variance into consideration, it has the most power. Since VAW-UniFrac has power similar to SqT, we only utilize UniFrac, W-UniFrac, and VAW-UniFrac in the following analyses of real data.

## Application 1: a study of bacterial communities on human skin across time after transplantation

We first studied the variable region 2 (V2) of bacterial 16S rRNA sequence data from Costello et al. [4] to understand the relationship between microbial communities in certain tissues after transplantation from another tissue. We present our results for the analysis of 80 microbial samples from four individuals (F2, F3, M1, M4), over two days, and with two plots by transplanting microbial organisms from the forehead to the left volar forearm at four different time points (0, 2, 4, 8 hours post-transplantation). The samples are listed in additional file 1.

We first assigned each sequence in samples to its closest relative in a phylogeny of the Greengenes core set [31] using BLAST's megablast [32] as in Hamady et al. [33]. Then we used only the phylogeny of the Greengenes core set and removed the leaves that were not involved in the comparison when comparing two samples. The Greengenes core set and the phylogeny were downloaded from the FastUnifrac website [33].

**Figure 1:**
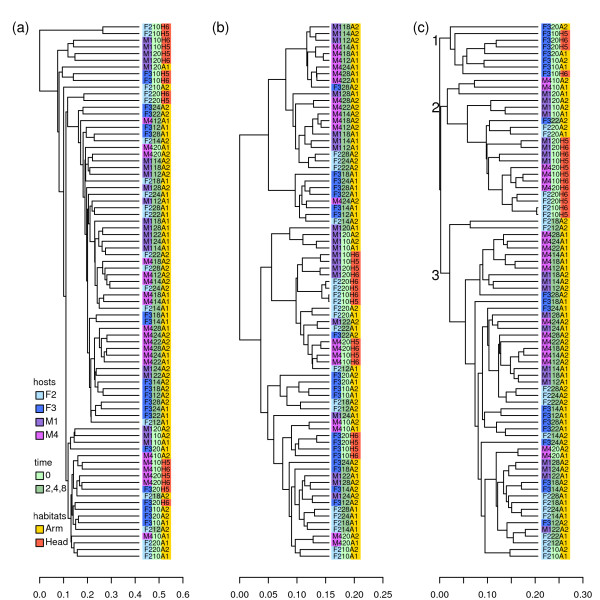
**Hierarchical clustering of 80 samples where the microbiotas from foreheads were transplanted to forearms**. UPGMA results using (a) UniFrac, (b) W-UniFrac and (c) VAW-UniFrac.

Figure 1 shows the UPGMA results of the 80 samples based on UniFrac, W-UniFrac and VAW-UniFrac, respectively. VAW-UniFrac clustered the 80 samples into three main clusters (Figure 1c). Cluster 1 contains 8 samples consisting of only native microbiotas from forehead and forearm of individual F3. Cluster 2 contains 21 samples, and 20 of them are native microbiotas from foreheads or forearms of other individuals in the experiment. The microbiotas from the foreheads form a tight subcluster of this cluster. Only one non-native microbiota (F322A2) belongs to this cluster and only four native microbiotas (M420A1, M420A2, F210A1, F210A2) are outside this cluster. In cluster 2, the samples from the same individual, same day and same site (forehead or forearm), but different plots, are clustered together almost perfectly. Cluster 3 contains 51 samples, and 47 of them are the microbiotas after inoculating microbiotas from the foreheads to the forearms. In this cluster, the samples collected at 2, 4 and 8 hours after inoculating at the same plot are always clustered with each other, indicating that the variation across time within the same plot is small compared to variation across different plots. In fact, the two plots "A1" and "A2" on forearm of one individual were always inoculated with microbiotas from foreheads of different individuals. The clustering is in accordance with the main conclusion of the original article: that the variation of skin bacterial communities is primarily explained by habitat, then by individual, and, finally, by time. Neither UniFrac nor W-UniFrac could obtain results as clear as VAW-UniFrac.

**Figure 2:**
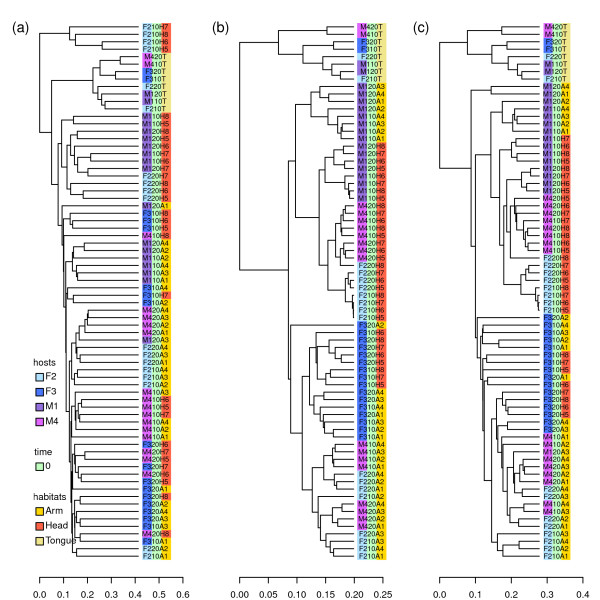
**Hierarchical clustering of 72 native microbiota samples from skin microbiota transplant experiments**. UPGMA results using (a) UniFrac, (b) W-UniFrac and (c) VAW-UniFrac.

We wondered why the 8 native samples from individual F3 were clustered together and separated from other native samples of the same habitats according to VAW-UniFrac, while the clustering of native samples from F3 was not detected at all by UniFrac and W-UniFrac. To investigate this phenomenon in more depth, we then applied the three statistics to all 72 native microbiotas in that study. The UPGMA results are shown in Figure 2. The results based on W-UniFrac and VAW-UniFrac are similar. According to both methods, the tongue samples are clustered separately from the skin samples. The forehead samples from individuals other than F3 are clustered together, while the forehead samples from F3 are separated from them. In the result derived by UniFrac, there is no such obvious clustering pattern, but the samples from forehead and forearm of F3 are always mixed. These results further support the observation that the forehead and forearm samples from F3 are significantly different from those of other individuals.

## Application 2: comparison of microbial communities in mouse gut

We then applied the three statistics to analyze the 16S rRNA sequences from mouse gut communities [2]. Lozupone et al. [17] applied UniFrac and W-UniFrac to this dataset and showed that analyses using the two different versions of UniFrac can lead to completely different conclusions. Therefore, we reanalyzed this dataset using VAW-UniFrac. We calculated the three statistics for each pair of the 19 communities and used hierarchical clustering and principal coordinate analysis (PCoA) to analyze the results. For each comparison, we used the same phylogenetic tree, but the leaves that were not in these two communities were removed so that the results of different comparisons were comparable.

**Figure 3:**
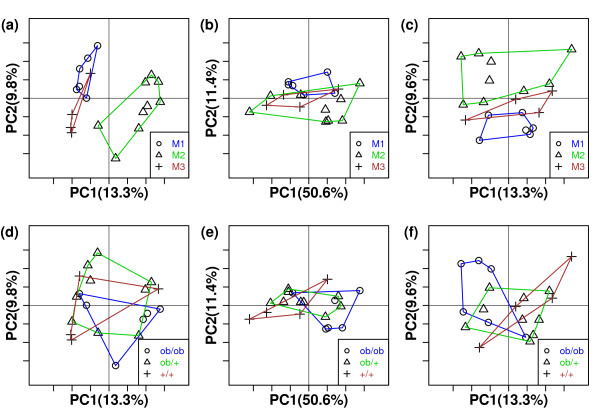
**PCoA plots of 19 microbial communities from mouse guts with three statistics**. (a through c) PCoA plots of 19 microbial communities from mouse guts with UniFrac, W-UniFrac and VAW-UniFrac, respectively, where communities are marked with different symbols according to families. (d through f) PCoA plots of 19 microbial communities from mouse guts with UniFrac, W-UniFrac and VAW-UniFrac, respectively, where communities are marked with different symbols according to genotypes. The first two principal coordinate axes in PCoA and percentages of variation that they explain are shown.

**Figure 4:**
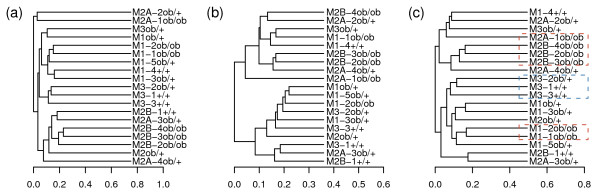
**Hierarchical clustering of 19 microbial communities from mouse guts with three statistics**. UPGMA clustering diagrams of 19 microbial communities from mouse guts with (a) UniFrac, (b) W-UniFrac and (c) VAW-UniFrac. The three mothers are found in sample M1, M2, and M3, and each offspring is named after its mother.

As observed in [17], both PCoA and UPGMA with UniFrac revealed clear associations between populations of microbial communities and kinship (Figure 3a and Figure 4a). The siblings were clustered together, including two mothers who were sisters (M1 and M3). M2A-1 and M2A-2, which were represented by fewer than 200 sequences, were the only mice not clustered with their mothers (Figure 4a), potentially resulting from the relatively small number of sequences in their communities. These results indicate that the presence/absence of microbial species in mouse gut is mainly determined by kinship. However, we were more interested in the performance of W-UniFrac and VAW-UniFrac. When W-UniFrac was used to cluster the mice, Lozupone et al. [17] indicated that there was a greater correlation with the obesity genotype than with kinship. Figure 3 shows that analysis using VAW-UniFrac reveals not only a correlation with obesity genotype, but also a clear correlation with kinship. Although PCoA analysis using W-UniFrac does not separate the kinship well, PCoA analysis using VAW-UniFrac clearly separates the offspring of M2 from the offspring of M1 and M3, indicating that kinship plays the most important role in gut microbial community (Figures 3b and 3c). In fact, the ob/ob individuals tend to cluster together within a given sibship using VAW-UniFrac, but this is not so clear by W-UniFrac. For instance, M1-1(ob/ob) and M1-2(ob/ob) clustered tightly by VAW-UniFrac (Figure 4c), but diverged by W-UniFrac (Figure 4b). Moreover, M3-1(+/+), M3-2(ob/+) and M3-3(+,+) were clustered together by VAW-UniFrac, but were dispersed by W-UniFrac (Figure 4b).

## Application 3: comparison of sequence collections derived by Sanger- and pyro-sequencing technologies

In order to see how the statistics perform when they are applied to sequence collections derived by different technologies, but from the same sample, we analyzed the 16S rRNA sequence data from soil and sediments from [25]. The same methods as in Application 1 were used to build the phylogeny of the sequences. Some short sequences that could not be assigned to any sequences in the Greengenes core set, most of which were less than 200 bp in length, were ignored in our analysis.

**Figure 5:**
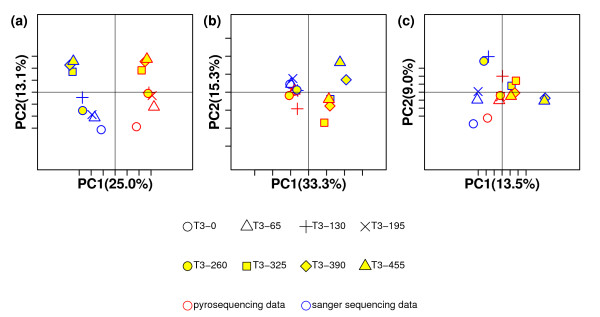
**PCoA plots of 16 sequence collections in Application 3 with three statistics**. PCoA plots of the 16 sequence collections with (a) UniFrac, (b) W-UniFrac and (c) VAW-UniFrac. The collections derived by pyrosequencing and Sanger sequencing from the same sample are represented by the same symbol with red and blue, respectively.

**Figure 6:**
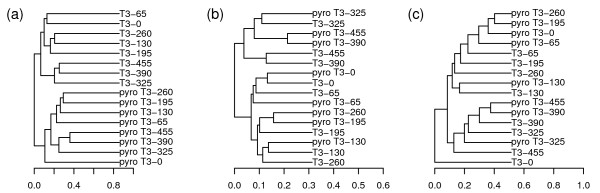
**Hierarchical clustering of 16 sequence collections in Application 3 with three statistics**. UPGMA clustering diagrams of the 16 sequence collections from 8 samples: T3-0, T3-65, T3-130, T3-195 T3-260, T3-325, T3-390, and T3-455 with (a) UniFrac, (b) W-UniFrac and (c) VAW-UniFrac. The Sanger sequencing data of T3-0 were named "T3-0", and the pyrosequencing data of T3-0 were named "pyro T3-0". Others were named in a similar manner.

After applying the three statistics to each pair of the 16 samples, we used PCoA and UPGMA to analyze the results and investigated the performance of the three statistics (Figure 5 and Figure 6). The results from both PCoA and UPGMA analysis indicated that UniFrac could not detect the similarity between two collections derived from the same sample. Instead, it clustered the data from Sanger sequencing and pyrosequencing separately. On the other hand, W-UniFrac and VAW-UniFrac could cluster some libraries of the two sequencing methods together according to geographical transect at some level, for example, the most water-logged sites (T3-325, T3-390, and T3-455). VAW-UniFrac separated T3-0 (the driest) from the others. This is reasonable because T3-0 was the only site in a vegetated, upland area, and other sites were from exposed lakebed and water's edge [25].

In fact, each pair of sequence libraries from the same sample using Sanger sequencing and pyrosequencing is very different because pyrosequencing detected a greater variety of low-abundance taxa compared to Sanger sequencing [25]. UniFrac emphasized those low-abundance taxa to a greater degree than the two weighted statistics. Consequently, UniFrac clustered the data from two techniques separately. However, the results from such an analysis can be misleading as pyrosequencing usually generates a very large number of sequences and tends to be more prone to error, while, on the other hand, the number of sequences from Sanger sequencing is usually relatively small, but tends to be more accurate. Sometimes, we hope samples from the same community cluster together irrespective of which sequencing technologies are used. Like when comparing communities based on sequence data from different studies, methods that are not highly sensitive to sequencing depth or sequencing technology are preferred. The weighted methods, such as W-UniFrac and VAW-UniFrac, are preferred in such cases.

## Application 4: analysis of compositions of tropical forests in central Panama

Although UniFrac and W-UniFrac were originally proposed to measure differences between microbial communities, they could also be applied to other communities, as long as the phylogeny of the individuals is available. Therefore, as another example, we applied the three statistics to tropical forest census data in three plots across a precipitation gradient in central Panama [26-28]. Different from the above applications, the original abundance information for each species present was available.

In order to obtain the phylogeny of tree species in these censuses, we referred to a dated phylogenetic tree of all angiosperm families [34]. It was downloaded from http://svn.phylodiversity.net/tot/megatrees/davies04.bl.new at the Phylomatic [35] website. There were 420 out of a total of 467 detectable species of the censuses included in this phylogeny. We reconstructed the tree by positioning the genera and species at 2/3 and 1/3 the age of the corresponding family, respectively, similar to [36].

**Figure 7:**
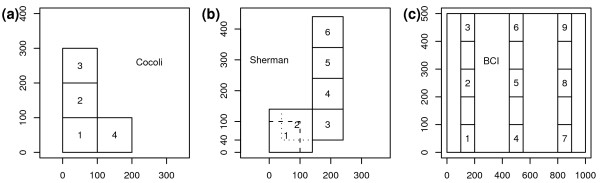
**Locations of the 19 1-ha tropical forest plots in Application 4**. (a) Cocoli, (b) Sherman, and (c) BCI. The three plots were divided into 19 1-ha plots according to topography and geographic coordinates, so that the different distances between the subdivided plots would help us to test the performance of the statistics.

**Figure 8:**
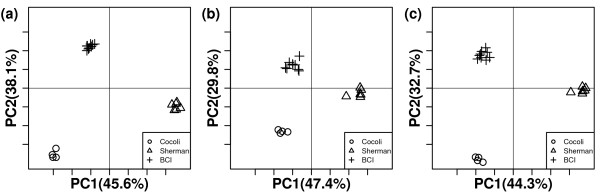
**PCoA results of 19 1-ha tropical forest plots in Application 4 with three statistics**. PCoA plots of 19 1-ha tropical forest plots of three sites using (a) UniFrac, (b) W-UniFrac, and (c) VAW-UniFrac. The first two principal coordinate axes in PCoA and percentages of variation that they explain are shown. Plots are denoted by different symbols according to sites.

**Figure 9:**
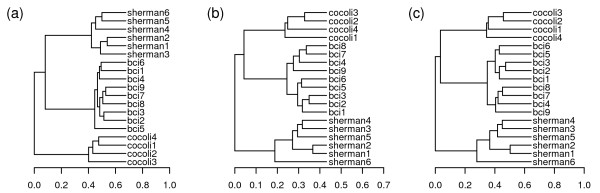
**Hierarchical clustering of 19 1-ha tropical forest plots in Application 4 with three statistics**. UPGMA diagrams of the 19 1-ha tropical forest communities with (a) UniFrac, (b) W-UniFrac and (c) VAW-UniFrac. The notations of the plots are the same as in Figure 7.

We divided these plots into 19 1-ha plots and compared their compositions using the three statistics. In order to obtain overall insight into the distribution of plants, trees from all census data were included in this study. Details about geographical division are shown in Figure 7. Both PCoA and UPGMA were used to analyze the results. PCoA plots showed that all three methods could perfectly cluster the communities by sites (Figure 8). This revealed that the tropical forests in the three sites differed substantially in species compositions, which had been verified by previous studies [1]. From UPGMA (Figure 9) clustering, we found that VAW-UniFrac and W-UniFrac provided almost identical clustering results.

Within-site comparisons had some interesting differences between UniFrac (Figure 9a) and its weighted variations (W-UniFrac and VAW-UniFrac, Figures 9b and 9c). For example, plot Sherman6 apparently stood apart from other Sherman plots based on the weighted measures. On the other hand, UniFrac separated Sherman1, 2, 3 from Sherman4, 5, 6. This revealed that the Sherman6 plot had a species composition similar to Sherman4, 5, but differed significantly in species abundance. The result is consistent with the fact that the Sherman6 plot was in a very young forest, probably cleared within the past 20 years [37]. The clustering results of 9 BCI plots also showed the superiority of the weighted measures. In Figures 9b and 9c, the clustering of the 9 small BCI plots is consistent with the geographic distributions of the plots(Figure 7). On the other hand, UniFrac separated plot BCI5 from the other BCI plots which seems not explicable. These resules indicate that within site, the differences between communities were mainly from abundances, while between sites, they were mainly from species presence/absence.

We also studied the effects of different tree construction methods for the sequences, e.g. neighbor-joining, maximum parsimony, and maximum likelihood, on the clustering results of the communities using the 16S rRNA sequence data in [25] as an example. The results are given in additional file 2. It is shown that the clustering of communities based on VAW-UniFrac does depend on the tree construction methods, however, the differences are generally small.

## Conclusions

In this paper, we studied UniFrac, a widely used phylogenetic method for comparing compositions of microbial communities, and a weighted variation, W-UniFrac, which takes abundance information into account. Both UniFrac and W-UniFrac can be written as a weighted sum of all the branch length in the phylogeny tree. However, different weighting methods resulted in differences in performance. For each branch, we showed that the number of sequences of one community that belong to the branch followed a hypergeometric distribution under the null hypothesis that community labels were not correlated with phylogeny. From this perspective, we developed a new variance adjusted weighted UniFrac that takes into account variation of the weights to test if two communities are different. Both simulations and applications on real data showed that VAW-UniFrac is more powerful than W-UniFrac. From real data analyses, we showed that our method could reveal biological insights not possible with either UniFrac or weighted UniFrac. Furthermore, our results supported the conclusion of Lozupone et al. [17] that the different versions of UniFrac can lead to different conclusions. With the increase of data containing abundance information, we expect that our new statistic will help to obtain new insights into community differences, especially for situations where the species are similar, but the differences in relative abundance are of great interest.

## Authors' contributions

YL and FS conceived and designed the experiments. QC performed the experiments and analyzed the data. QC drafted the manuscript and YL and FS finalized the manuscript. All authors read and approved the final manuscript.

## Supplementary Material

Additional file 1 **The 80 samples where the microbiotas from foreheads were transplanted to forearms in Application 1**. It contains the individuals, days, plots, and the times of sampling the communities. Click here for file

Additional file 2 **The effects of tree-construction methods on the VAW-UniFrac results**. Four tree construction methods are used to build a tree for the sequences. These methods include: 1) neighbor-joining, 2) maximum parsimony, 3) maximum likelihood, and 4) BLAST assignments of the sample sequences to the closet relatives on a known phylogenetic tree. The effects of the different tree construction methods on the results of UniFrac, W-UniFrac, and VAW-UniFrac are presented. Click here for file
