# Supplementary material for: Variance adjusted weighted UniFrac: a powerful beta diversity measure for comparing communities based on phylogeny
Source: BMC Bioinformatics. 2011 Apr 25;12:118. doi: 10.1186/1471-2105-12-118 (PMC3108311; doi:10.1186/1471-2105-12-118)
Supplement: Additional file 1 — The 80 samples where the microbiotas from foreheads were transplanted to forearms in Application 1. It contains the individuals, days, plots, and the times of sampling the communities. [file 1471-2105-12-118-S1.PDF]

Additional Table 1 - The 80 samples where the microbiotas from foreheads were transplanted to forearms in Application 1 of the main text.

Forearm(forehead transplant)					
individual,day,plot	foreign microbiota	0hrs	2hrs	4hrs	8hrs
F2, Day1, PlotA1	F210H5	F210A1	F212A1	F214A1	F218A1
F2, Day1, PlotA2	F310H6	F210A2	F212A2	F214A2	F218A2
F2, Day2, PlotA1	F220H5	F220A1	F222A1	F224A1	F228A1
F2, Day2, PlotA2	M120H6	F220A2	F222A2	F224A2	F228A2
F3, Day1, PlotA1	F310H5	F310A1	F312A1	F314A1	F318A1
F3, Day1, PlotA2	F210H6	F310A2	F312A2	F314A2	F318A2
F3, Day2, PlotA1	F320H5	F320A1	F322A1	F324A1	F328A1
F3, Day2, PlotA2	M420H6	F320A2	F322A2	F324A2	F328A2
M1, Day1, PlotA1	M110H5	M110A1	M112A1	M114A1	M118A1
M1, Day1, PlotA2	M410H6	M110A2	M112A2	M114A2	M118A2
M1, Day2, PlotA1	M120H5	M120A1	M122A1	M124A1	M128A1
M1, Day2, PlotA2	F220H6	M120A2	M122A2	M124A2	M128A2
M4, Day1, PlotA1	M410H5	M410A1	M412A1	M414A1	M418A1
M4, Day1, PlotA2	M110H6	M410A2	M412A2	M414A2	M418A2
M4, Day2, PlotA1	M420H5	M420A1	M422A1	M424A1	M428A1
M4, Day2, PlotA2	F320H6	M420A2	M422A2	M424A2	M428A2
