# Supplementary material for: Variance adjusted weighted UniFrac: a powerful beta diversity measure for comparing communities based on phylogeny
Source: BMC Bioinformatics. 2011 Apr 25;12:118. doi: 10.1186/1471-2105-12-118 (PMC3108311; doi:10.1186/1471-2105-12-118)
Supplement: Additional file 2 — The effects of tree-construction methods on the VAW-UniFrac results. Four tree construction methods are used to build a tree for the sequences. These methods include: 1) neighbor-joining, 2) maximum parsimony, 3) maximum likelihood, and 4) BLAST assignments of the sample sequences to the closet relatives on a known phylogenetic tree. The effects of the different tree construction methods on the results of UniFrac, W-UniFrac, and VAW-UniFrac are presented. [file 1471-2105-12-118-S2.PDF]

Additional File 2: The effects of tree-construction methods on the VAW-UniFrac results

For all the three methods studied in this paper, UniFrac, weighted UniFrac (W-UniFrac) and variance adjusted weighted UniFrac (VAW-UniFrac), a phylogenetic tree of the sequences from the communities is needed. Many different tree construction methods are available including maximum parsimony, distance based methods such as neighbor-joining, and maximum likelihood trees. A natural question is how the different tree-constructing methods affect the subsequent analysis? Lozupone et al.[1] studied the effects of seven tree construction methods on UniFrac and W-UniFrac. They showed that the results from both UniFrac and W-UniFrac are robust to different tree-constructing methods. To investigate the effects of various tree-constructing methods on VAW-UniFrac, we applied four different tree-constructing methods to eight 16S rRNA gene sequence data sets (the Sanger sequencing data sets in Application 3 [2]) and used principal coordinates analysis (PCoA) [3] to show the results. The data sets were described in the main text.

1 Methods

Tree-constructing methods

We chose four tree construction methods as examples to study their effects on the VAW-UniFrac results. These methods include: 1) neighbor-joining, 2) maximum parsimony, and 3) maximum likelihood [4]. The program ClustalX 2.1 [5] was used to align the sequences. The resulting trees were rooted by the RETREE program in PHYLIP 3.69 [6] using the midpoint rooting method. The fourth method [7] is based on BLAST assignment of the sample sequences to the closet relatives on a known phylogenetic tree. This method was used in the main text and it uses a given phylogeny as guide instead of building a tree *de novo*.

- The **neighbor-joining method** was implemented using the “Neighbor-Joining” in the NEIGHBOR program in PHYLIP 3.69, based on the

distance matrix generated by the DNADIST program in PHYLIP 3.69 under the F84 nucleotide substitution model.

- The **parsimony method** was implemented using the DNAPARS program in PHYLIP 3.69. The first tree in the resulting trees tied for optimal score was used as a representative for the maximum parsimony tree.
- The **maximum likelihood method** was implemented using the DNAML program in PHYLIP 3.69, which uses a hidden Markov model to infer different evolution rates at different sites.
- For the **BLAST-based sequence assignment method**, each sample sequence was assigned to its closest relative in a phylogeny of the Greengenes core set [8] using BLAST's megablast [9] as in Hamady et al. [7]. We then used only the phylogeny of the Greengenes core set and removed the leaves that were not involved in the comparison when comparing two communities. The Greengenes core set and the phylogeny were downloaded from the FastUnifrac website [7].

2 Results

After constructing phylogenic trees using the four methods, we applied UniFrac, W-UniFrac, and VAW-UniFrac to each phylogeny to compare the 8 samples. We then used principal coordinates analysis (PCoA) [3] to show the results. The PCoA plots of the 8 samples using the neighbor-joining, maximum parsimony, maximum likelihood, and the BLAST-based sequence assignment methods are shown by Additional figures 1, 2, 3, and 4, respectively.

Additional figure 1: The PCoA plots of the 8 samples using (A)UniFrac, (B)W-UniFrac, and (C) VAW-UniFrac based on the tree constructed by the neighbor-joining method.

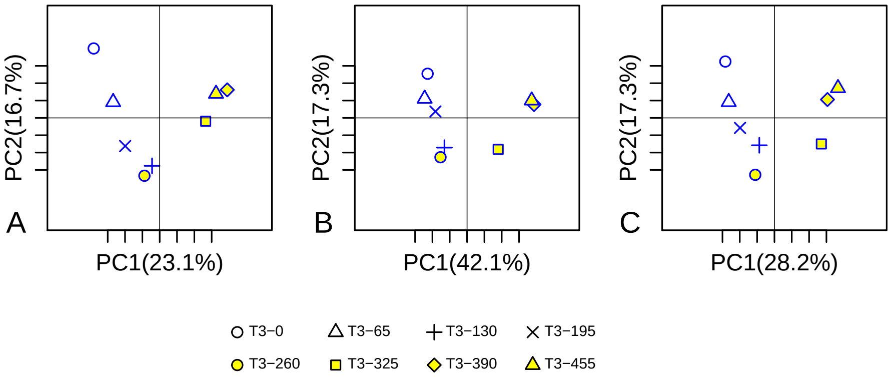

Additional figure 2: The PCoA plots of the 8 samples using (A)UniFrac, (B)W-UniFrac, and (C) VAW-UniFrac based on the tree constructed by the parsimony method.

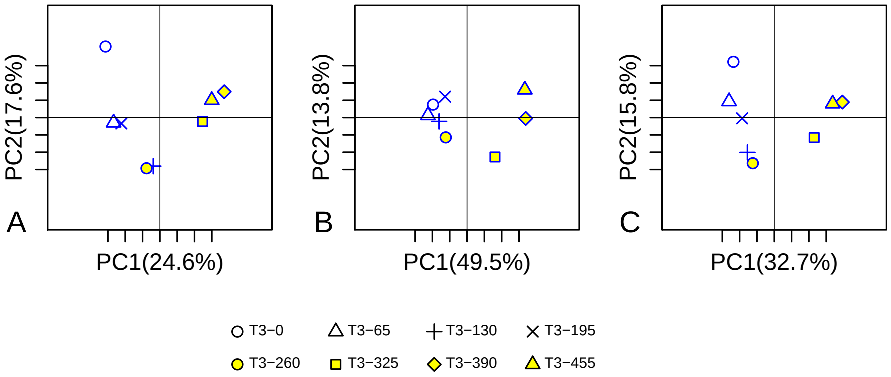

Additional figure 3: The PCoA plots of the 8 samples using (A)UniFrac, (B)W-UniFrac, and (C) VAW-UniFrac based on the tree constructed by the maximum likelihood method.

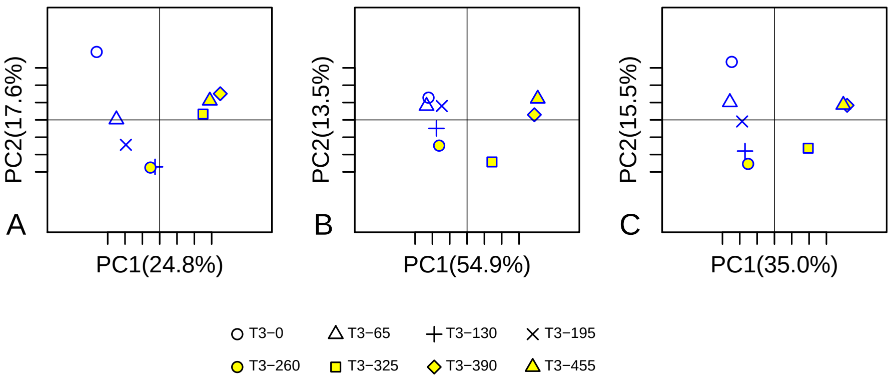

Additional figure 4: The PCoA plots of the 8 samples using (A)UniFrac, (B)W-UniFrac, and (C) VAW-UniFrac based on the tree constructed by the BLAST-based sequence assignment method.

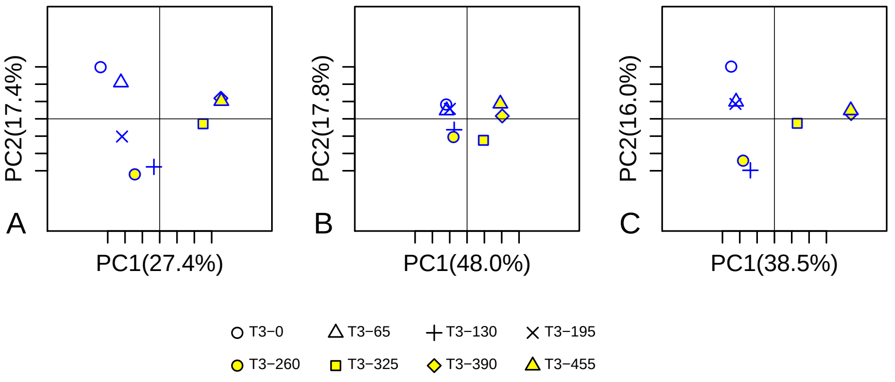

From these figures, we can see that the different tree-constructing methods indeed affect the results, but the effects are small. For the four different tree-constructing methods, the PCoA results are generally similar.

References

[1] Lozupone CA, Hamady M, Kelley ST, Knight R: **Quantitative and qualitative β diversity measures lead to different insights into factors that structure microbial communities**. *Appl. Environ. Microbiol.* 2007, **73**:1576–1585.

[2] Hollister E, Engledow A, Hammett A, Provin T, Wilkinson H, Gentry T: **Shifts in microbial community structure along an ecological gradient of hypersaline soils and sediments**. *ISME J.* 2010, **4**:829–838.

[3] Gower JC: **Some distance properties of latent root and vector methods used in multivariate analysis**. *Biometrika* 1966, **53**:325–338.

[4] Felsenstein J: *Inferring phylogenies*. Sunderland, MA: Sinauer Associates, Inc 2004.

[5] **CLUSTAL** [http://www.clustal.org/].

[6] **PHILIP** [http://evolution.gs.washington.edu/phylip.html].

[7] Hamady M, Lozupone C, Knight R: **Fast UniFrac: facilitating high-throughput phylogenetic analyses of microbial communities including analysis of pyrosequencing and PhyloChip data**. *ISME J.* 2010, **4**:17–27.

[8] DeSantis T, Hugenholtz P, Larsen N, Rojas M, Brodie E, Keller K, Huber T, Dalevi D, Hu P, Andersen G: **Greengenes, a chimera-checked 16S rRNA gene database and workbench compatible with ARB**. *Appl Environ Microbiol* 2006, **72**:5069–5072.

[9] Altschul S, Gish W, Miller W, Myers E, Lipman D: **Basic local alignment search tool**. *J Mol Biol* 1990, **215**:403–410.

[10] Lozupone C, Knight R: **UniFrac: a new phylogenetic method for comparing microbial communities**. *Appl. Environ. Microbiol.* 2005, **71**:8228–8235.
